# A Unique Time-Reversal Algorithm-Enabled Flexible Ultrasound Transducer with a Controllable Acoustic Field

**DOI:** 10.3390/s24175635

**Published:** 2024-08-30

**Authors:** Lu Jia, Yingzhan Yan, Jing Xu, Yuan Gao

**Affiliations:** 1 Information Science Academy, China Electronics Technology Group Corporation, Beijing 100142, China; jialu199408@163.com (L.J.); yyz712@163.com (Y.Y.); xujing_1999@126.com (J.X.); 2 National Key Laboratory of Integrated Circuits and Microsystems, Beijing 100142, China

**Keywords:** flexible ultrasonic devices, phased-array transducer, time-reversal algorithm, controllable acoustic field

## Abstract

Flexible ultrasonic devices represent a feasible technology for providing timely signal detection and even a non-invasive disease treatment for the human brain. However, the deformation of the devices is always accompanied by a change in the acoustic field, making it hard for accurate focusing. Herein, we report a stable and flexible transducer. This device can generate a high-intensity acoustic signal with a controllable acoustic field even when the device is bent. The key is to use a low-impedance piezoelectric material and an island-bridge device structure, as well as to design a unique time-reversal algorithm to correct the deviation of signals after transcranial propagation. To provide an in-depth study of the acoustic field of flexible devices, we also analyze the effects of mechanical deformation and structural parameters on the corresponding acoustic response.

## 1. Introduction

The fast-growing interest in the Internet of Things, artificial intelligence, soft robotics, and personal health care has promoted the development of flexible and wearable electronic devices [1,2,3,4,5,6,7,8,9]. So far, flexible sensing devices that can detect optical signals [1], strain [2,3], and electrochemical signals [6] are mostly in the form of wearable accessories or skin patches. These devices can only obtain biomedical information from human skin, superficial blood vessels, or bodily fluids, but they can hardly collect information from deep organs (e.g., the detection of brain signals). The design of implantable sensing devices can enable deep signal detection from the human body [10,11]. However, these implant devices must fulfill stringent requirements for excellent biocompatibility, and the implant surgery is mostly painful. Particularly when detecting brain signals, the implant surgery may lead to secondary infections during device implantation and removal, which seriously harm the human body.

Ultrasonic devices appear to be promising candidates to address this challenge because acoustic waves can penetrate crucial internal organs and tissues and remain harmless to the human body if the signal intensity is not too high. As such, the use of ultrasonic devices can provide timely signal detection [12,13,14,15] and even non-invasive disease treatment of the human brain [16,17,18,19,20]. In order to obtain stable detection of signals, high-performance and flexible ultrasonic devices, which can conformally contact and closely adhere to the rigid or irregular surfaces of organs, are highly sought after. To date, almost all studies of flexible ultrasonic devices have focused on either the synthesis of flexible ultrasonic materials or the design of flexible device structures. Among them, the fabrication of a phased-array transducer using piezoelectric materials [21,22] (e.g., polyvinylidene fluoride and its copolymers) and an “island-bridge” device structure [14,23,24,25,26] is a promising approach to develop ultrasonic devices with both high performance and prominent flexibility. These devices have been successfully applied in biomedical fields such as real-time monitoring of blood flow [27], deep tissue sensing [12,15], cardiac pacing [28], treatment of brain diseases [19,20], etc.

It is noted that, for diseases such as epilepsy and Parkinson’s, sudden or long-term neuroregulatory therapy is often needed. The reported ultrasonic neuroregulatory systems can help provide timely and effective intervention and treatment for these diseases [18,19]. However, a common issue with these devices resides in the reflection or absorption of acoustic signals by the organs of animals/humans (especially the skull), leading to a drastic decrease in signal intensity. Additionally, the deformation of the devices is always accompanied by changes in the acoustic field, making it hard to precisely monitor the propagation direction of the acoustic signals.

Herein, we report a flexible phased-array transducer, which can generate a high-intensity acoustic signal with traceable signal propagation even when the device is bent. The key is to use a thin and low-impedance piezoelectric composite as the transducer material and to design a unique algorithm to correct the deviation of the signals after transcranial propagation. To provide an in-depth study of the acoustic field of the flexible devices, we also analyze the effects of mechanical deformation and structural parameters on the corresponding acoustic response using FEA.

## 2. Design and Simulation

**Figure 1:**
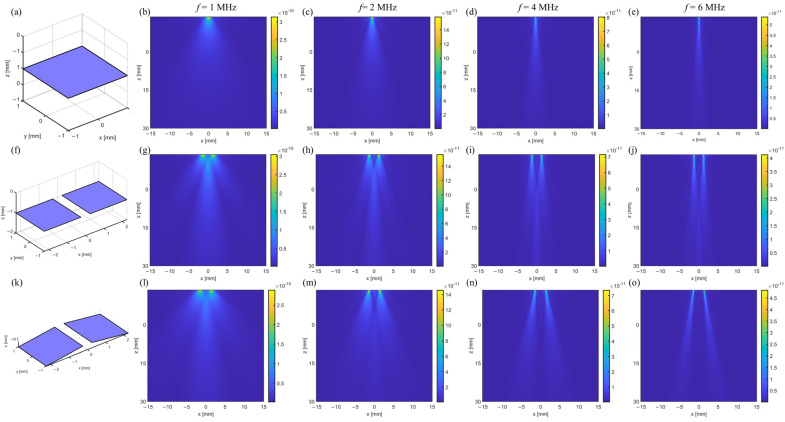
Steady-state emission acoustic field of the flexible transducer under different conditions. (**a**) A single-pixel transducer. The pixel possesses a square geometry with a side length of 2 mm. (**b**–**e**) The emission acoustic field of a single-pixel transducer at the frequencies of 1 MHz, 2 MHz, 4 MHz, and 6 MHz, respectively. (**f**) A dual-pixel transducer w/o bending. The distance between neighboring pixels is 0.5 mm. (**g**–**j**) The emission acoustic field of a dual-pixel transducer at the frequencies of 1 MHz, 2 MHz, 4 MHz, and 6 MHz, respectively. (**k**) A flexed dual-pixel transducer with the bending radius of 30 mm. (**i**–**o**) The emission acoustic field of a flexed dual-pixel transducer with the bending radius of 30 mm at the frequencies of 1 MHz, 2 MHz, 4 MHz, and 6 MHz, respectively.

To guide the design and fabrication of desirable ultrasonic devices, we first simulate the emission acoustic field of the transducer with a single pixel or multiple pixels using Field II in MATLAB R2021b. To reduce the boundary effects of the signals during the simulation, a sinusoidal signal modulated with a Hanning window is used to stimulate the transducer. The pixel possesses a square geometry with a side length of 2 mm. The distance between neighboring pixels is 0.5 mm. Figure 1a–e illustrate the steady-state emission acoustic field of a single-pixel transducer at various frequencies. With the increase in frequency, the intensity of the acoustic signal is enhanced in the forward direction and decreased in the lateral direction, indicating an improved propagation direction of the acoustic field. The corresponding acoustic field of a dual-pixel transducer is shown in Figure 1f–j. Although the intensity of the acoustic signals is enhanced with the increase in frequency, the converged acoustic beam gradually splits into two separate beams at a frequency of 6 MHz. This result differs from that of a single-pixel transducer, which can be attributed to the superposition of signals from different pixels.

The conformal contact between the flexible transducer and the surface of the human brain enables better acoustic signal control and detection. In this scenario, the desirable transducers should possess a high tolerance to deformation, while maintaining a stable acoustic field. We thus further simulate the acoustic field of the dual-pixel transducer when bent at a radius of 30 mm, as shown in Figure 1k–o. The flexed transducer shows a relatively clear main lobe at frequencies of 1 MHz and 2 MHz. When compared to the flat transducer, the bent design of the device has a negligible effect on its acoustic field at low frequencies. As such, we believe that the transducer made with an array structure and stimulated at low frequencies (e.g., 2 MHz) can be tolerant to deformation and show a stable acoustic field.

**Figure 2:**
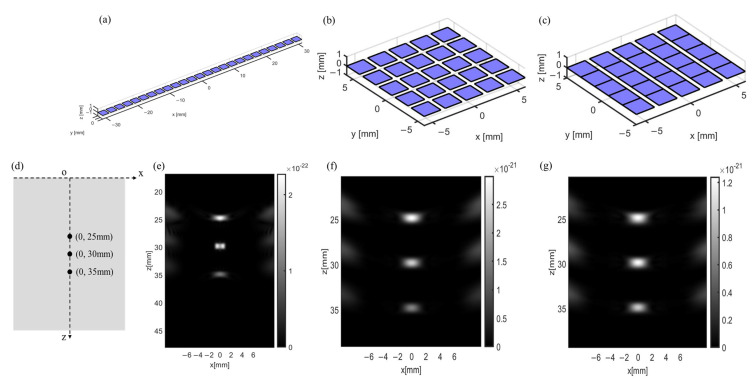
Different arrangement of 25 pixels in the phased-array transducers and their corresponding echo imaging. (**a**) A linear array. (**b**) A square array. Each pixel in the square array can work individually. (**c**) A multirow array. It consists of five individual lines and each line is made of five close-connected pixels. (**d**–**g**) Echo imaging is generated by a linear array, a square array, and a multirow array at three observed points.

The acoustic field of a transducer can also be affected by the arrangement of its pixels. To verify this statement, 25 pixels are aligned in a linear array, a square array, and a multirow array, as shown in Figure 2a–c. For the linear and square arrays, each pixel is electrically connected with serpentine metal wire and can work individually. To simplify the simulation model, a multirow array consisting of five individual lines is used, with each line comprising five pixels that connect closely w/o using metal wires. All the arrays possess the same focusing point located below the central pixel with a distance of 30 mm. We then compare the echo imaging of artificial phantoms generated by these flexible transducers at three points, including 0, 25 mm, 0, 30 mm, and 0, 35 mm, as shown in Figure 2d–g. According to the simulation results, the echo imaging generated by the square array and the multirow array is similar at all three observed points. Both arrays exhibit better focused acoustic signals than the linear array, as demonstrated by the smaller spot size at the focusing point. In addition, the square array and multirow array show a higher signal intensity (~10 times) than that of the linear array at the three observed points under the same conditions, which further proves that a desirable acoustic field of the transducer can be achieved with the design of either a square or multirow array. Considering the practical application of brain disease detection or treatment, the multirow array design requires fewer flexible interconnections among each pixel than the square array, benefiting the fabrication of transducers with smaller future sizes. In this scenario, we regard the multirow array as the optimal transducer design in this work.

**Figure 3:**
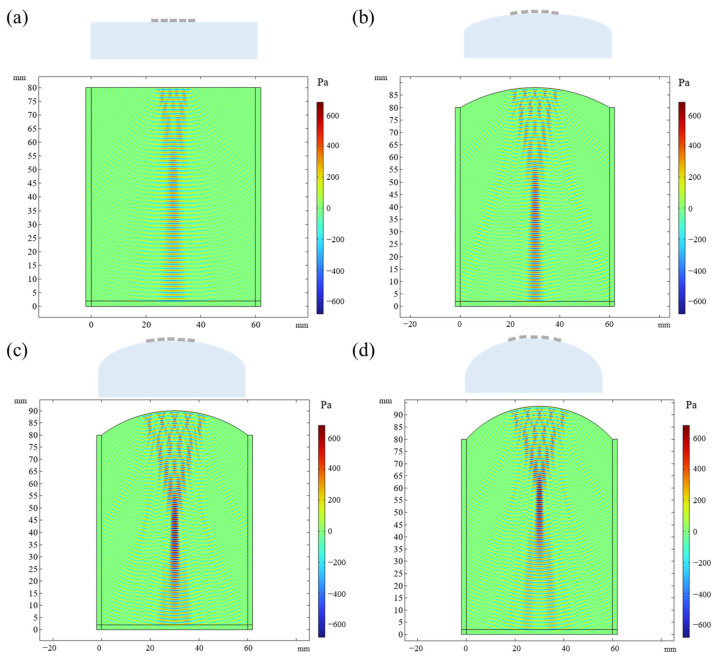
The evolution of acoustic field when the phased-array transducer is bent under different bending conditions. (**a**) Flat condition w/o bending. (**b**) Bending at a curvature of 16.7 mm^−1^. (**c**) Bending at a curvature of 20 mm^−1^. (**d**) Bending at a curvature of 25 mm^−1^.

We also simulate the evolution of the acoustic field when the multirow array is bent under different bending conditions. Under flat conditions, the acoustic waves generated by different pixels propagate vertically and result in a converged acoustic beam with a uniform energy distribution along the propagation direction, as shown in Figure 3a. When the array is bent, the acoustic waves can also focus on a certain point right below the center of the array, and the distance from the focal point to the array can be reduced with an increase in bending curvature, as shown in Figure 3b,c. It should be noted that at a large bending curvature, e.g., 25 mm^−1^, the energy of the acoustic wave attenuates after the focal point because of the large diffusion angle, which negatively affects the propagation distance of the acoustic waves, as shown in Figure 3d.

**Figure 4:**
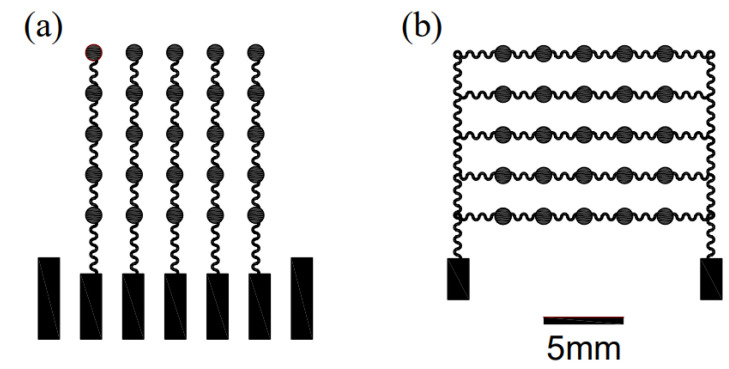
Schematic image of the designed flexible phased-array transducer. (**a**) Top electrodes. (**b**) Bottom electrodes.

Based on the above simulation results, we then design a flexible transducer. In this transducer, each pixel possesses a geometry size of 2 mm × 2 mm × 1 mm and is bridged by the serpentine interconnects, as shown in Figure 4. A bulk 1–3 piezoelectric composite with a thickness of 1 mm and a high resonant frequency of 2 MHz is used as the active material because of its multiple appealing properties. Firstly, the 1–3 piezoelectric composite exhibits a lower sound impedance of 15 MRal than traditional PZT materials (30 MRal), which is in favor of a better matched impedance with the organs of the animal/human tissues (e.g., 2 MRal for the skulls). The matched impedance can reduce the signal reflection between the devices and organs, hence ensuring a high energy retention of the acoustic signals. Additionally, benefiting from the unique material structure, 1–3 piezoelectric composite materials exhibit a suppressed transverse vibration when subjected to external stimulation so as to achieve better control of the acoustic signal.

## 3. Fabrication Process and Performance Testing

**Figure 5:**
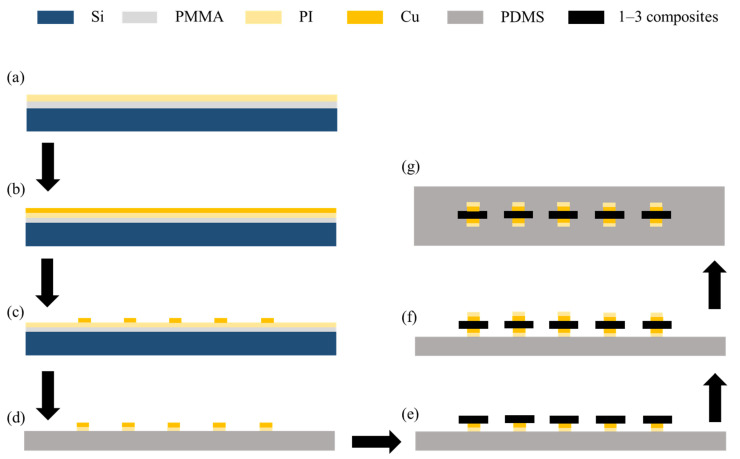
Fabrication of a phased-array transducer. (**a**) Deposition of polymethyl methacrylate (PMMA) and polyimide (PI) onto silicon substrates via spin coating. (**b**) Deposition of copper (Cu) thin films onto PI via evaporation. (**c**,**d**) Fabrication of patterned Cu electrodes via reactive ion etching (RIE) and the removal of excess PI. (**e**,**f**) Connection of each piezoelectric composite pixel to the bottom and top Cu electrode with silver paste. (**g**) Encapsulation of transducer with polydimethylsiloxane (PDMS).

**Figure 6:**
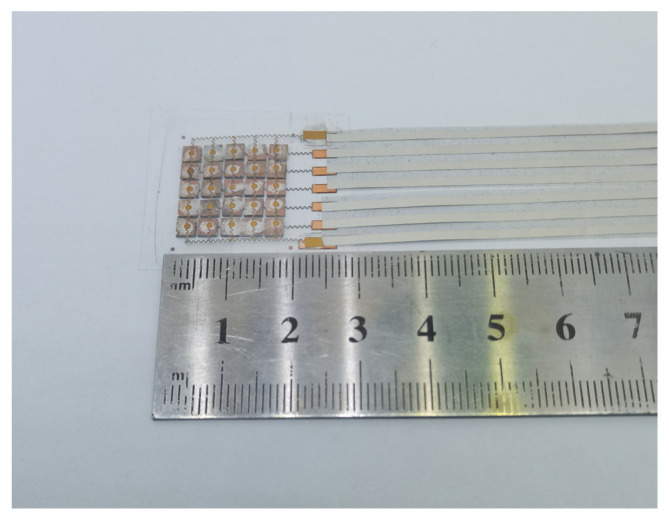
Image of the phased-array transducer.

The fabrication of a phased-array transducer and the resulting device image are shown in Figure 5 and Figure 6. Typically, a layer of polymethyl methacrylate (PMMA) is spin-coated on a silicon wafer, followed by the coating of another polyimide (PI) layer on the PMMA. In this scheme, PI serves as a protective layer, which can relieve the strain mismatch among different layers. To achieve flexible interconnects, a layer of copper (Cu) is evaporated on the PI and etched into targeted patterns. Afterwards, the excess PI is removed via reactive ion etching (RIE). The top or bottom serpentine Cu electrodes are achieved after transferring the patterned Cu layer onto a thin PDMS film. The 1–3 piezoelectric composite, serving as the pixel of the transducer, is sandwiched between the top and bottom Cu electrodes and further bonded by the silver paste. In this device, ACF lines are used as the readout circuit to collect the signals from individual pixels. At last, another polydimethylsiloxane (PDMS) layer is used to encapsulate the whole device, ensuring the good electrical properties and flexibility of the device.

**Figure 7:**
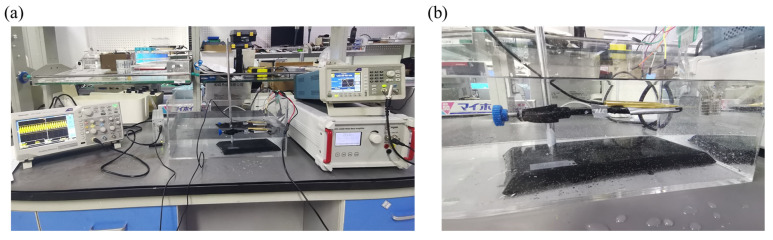
Setup used for testing the acoustic performance of phased-array transducer. (**a**) Image of the whole testing setup. (**b**) Zoomed-in image of phased-array transducer under test.

The good reliability of the phased-array transducer can be reflected by the performance consistency of its channel. Typically, the resonant frequency and the intensity of the acoustic signals are two important performance parameters for transducers. Figure 7 displays the setup used for testing the acoustic performance of the as-fabricated transducer. During the test, a sinusoidal wave is generated by a generator and amplified by a power amplifier, which can be used as the stimuli signal. The response signal produced by the transducer is collected by a highly sensitive hydrophone and then can be visualized via an oscilloscope. To mimic the environment of the skull, the acoustic performance of the phased-array transducer is tested in a tank filled with water.

**Figure 8:**
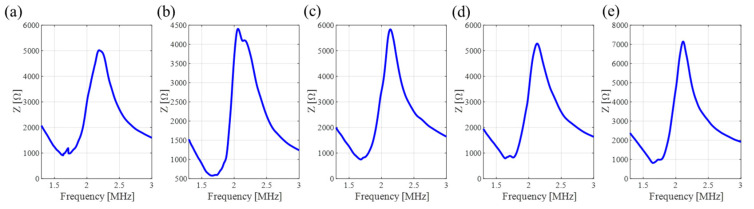
Test of resonant frequency of each channel. (**a**) Channel 1. (**b**) Channel 2. (**c**) Channel 3. (**d**) Channel 4. (**e**) Channel 5.

Five channels are tested using an impedance analyzer. The resonance frequency of each channel exhibits a similar value of around 2 MHz, as shown in Figure 8. The sound pressure shows a linear relationship with the intensity of the acoustic signals, which can be calculated using the following formula:

*P* = *Iε* (1)

where *P* represents the effective sound pressure, *ε* is the sensitivity of the hydrophone at 2 MHz, and I is the intensity of the signal. According to Table 1, each pixel renders a similar sound pressure of ~36 kPa. Both the similar resonant frequency and the sound pressure indicate that each pixel possesses a good consistency of acoustic performance.

## 4. Transcranial Focused Ultrasound Simulation

It is noted that the transducer should be able to generate a high-intensity acoustic signal, which can overcome the high blood–brain barrier permeability. That is to say, the acoustic intensity should be higher than 150 kPa if the transducer is used for brain signal detection and treatment in mice [29]. Considering that the phased-array transducer consists of 25 pixels, the device can theoretically generate an acoustic signal with an overall signal intensity of ~900 kPa.

For brain signal detection or disease treatment, the focus (where the disease resides inside the skull) and the position of a transducer should be predefined, so that the acoustic intensity and the propagation can be regulated accordingly with a specific algorithm. The use of a phased-array transducer with multiple pixels can further enhance the accuracy of acoustic signal detection and regulation as well as provide good flexibility. However, the levels of deformation for each pixel in a phased-array transducer are different, which generates a time difference for ultrasound propagation, thus making it hard for accurate focusing.

**Figure 9:**
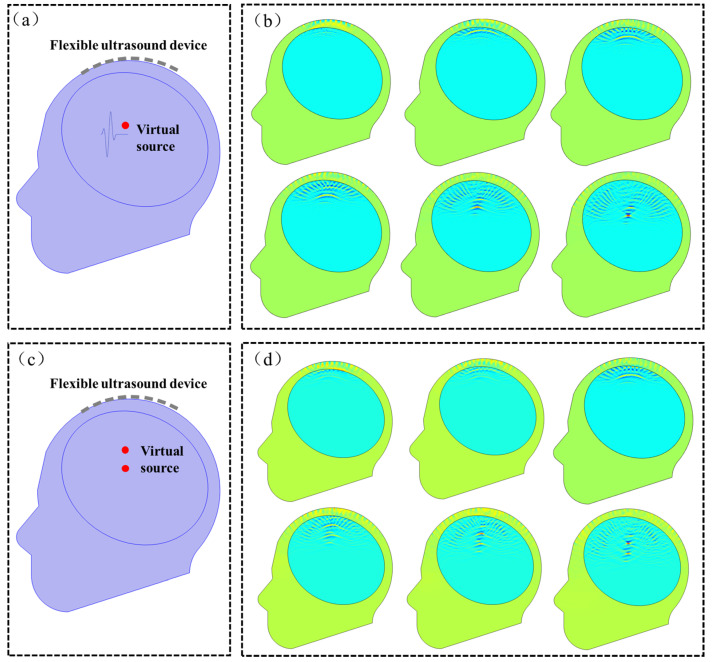
Simulation study for the acoustic field at the interface between the skull and the phased-array transducer in single and two virtual points. (**a**,**b**) Geometric model and snapshots of the acoustic field at different times in a single virtual point. (**c**,**d**) Geometric model and snapshots of the acoustic field at different times in two virtual points.

To tackle this issue, we propose a proof-of-concept algorithm, namely the Virtual Point Source Time Reversal Algorithm (VPSTRA), which can compensate for time differences in ultrasound transmission through the skull. The time-reversal method is one of the most widely used methods in brain-focusing therapy [30,31,32]. With the development of computing technology, high-precision numerical simulation methods for acoustic fields can be used to simulate the time-reversal process based on a “virtual source” on a computer, achieving truly non-destructive and precise focusing of transcranial ultrasound. Compared to traditional algorithms, this method can overcome the non-uniformity of skull bones and the difficulty in ultrasound focusing caused by the deformation of pixels in the phased-array transducer. The design principle of VPSTRA is shown in Figure 9a and can be illustrated as follows: Firstly, we define a virtual point source (marked with a red dot) inside the skull and ensure that each pixel of the transducer can receive signals from this point. Since the time for the signal that propagates from the virtual point to each pixel is different, we thus eliminate the time differences by using the time-reversal algorithm. Finally, we use the corrected signals as the stimuli for each pixel so as to achieve focal reformation at the virtual point.

To demonstrate the feasibility of VPSTRA, we simulate the change in the acoustic field at the interface between skulls and the transducers for both a single virtual point and two virtual points, shown in Figure 9. The acoustic speeds in the skull and other parts are set as 3360 m s^−1^ and 1500 m s^−1^, respectively. The densities of the skull region and other parts are 1658 kg m^−3^ and 1000 kg m^−3^, respectively. Using the corrected signals as stimuli, the acoustic field of the phased-array transducer shows a good focal formation both in single and two virtual source points in Figure 9.

## 5. Conclusions

This paper reported a stable and flexible transducer consisting of 25 pixels, in which a low-impedance piezoelectric material and an island-bridge device structure were used. Ultrasonic emission experiments were carried out in water to research the ultrasonic output of a flexible device. The measured intensity of each single element was around 36 kPa. A unique Virtual Point Source Time Reversal Algorithm (VPSTRA) was proposed to correct the deviation in the signals after transcranial propagation. The flexible phased-array transducer design, together with VPSTRA, can simultaneously reduce the future size of the transducer, avoid the use of hard materials, and resolve the difficulty in device fixation, which greatly facilitates the application of ultrasound methods in the treatment of brain diseases.

## Figures and Tables

**Table 1:** Intensity of the acoustic signal for one pixel in each channel.

	Channel	1	2	3	4	5
Excitation Voltage						
10 V		35.46 kPa	36.76 kPa	35.86 kPa	37.23 kPa	36.79 kPa

## Data Availability

The data presented in this study are available on request from the corresponding author.
